# PCBP-1 regulates alternative splicing of the CD44 gene and inhibits invasion in human hepatoma cell line HepG2 cells

**DOI:** 10.1186/1476-4598-9-72

**Published:** 2010-04-02

**Authors:** Tong Zhang, Xian-Hong Huang, Lan Dong, Deqing Hu, Changhui Ge, Yi-Qun Zhan, Wang-Xiang Xu, Miao Yu, Wei Li, Xiaohui Wang, Liujun Tang, Chang-Yan Li, Xiao-Ming Yang

**Affiliations:** 1 Beijing Institute of Radiation Medicine, Beijing, 100850, China; 2 Tianjin University, Department of Pharmaceutical Engineering, Tianjin 300072, China; 3 State Key laboratory of Proteomics, Beijing Proteome Research Center, Beijing 102206, China; 4 Anhui Medical University, Hefei 230032, China; 5 Department of Anesthesiology, General Hospital of Chinese People's Armed Police Forces, Beijing 100039, China

## Abstract

**Background:**

PCBP1 (or alpha CP1 or hnRNP E1), a member of the PCBP family, is widely expressed in many human tissues and involved in regulation of transcription, transportation process, and function of RNA molecules. However, the role of PCBP1 in CD44 variants splicing still remains elusive.

**Results:**

We found that enforced PCBP1 expression inhibited CD44 variants expression including v3, v5, v6, v8, and v10 in HepG2 cells, and knockdown of endogenous PCBP1 induced these variants splicing. Invasion assay suggested that PCBP1 played a negative role in tumor invasion and re-expression of v6 partly reversed the inhibition effect by PCBP1. A correlation of PCBP1 down-regulation and v6 up-regulation was detected in primary HCC tissues.

**Conclusions:**

We first characterized PCBP1 as a negative regulator of CD44 variants splicing in HepG2 cells, and loss of PCBP1 in human hepatic tumor contributes to the formation of a metastatic phenotype.

## Background

Alternative pre-mRNA splicing is emerging as an important mechanism of genetic diversity. Microarray data show that 74% of human genes undergo alternative splicing, which generates different protein isoforms. Alternative splicing of several genes has been implicated in tumorigenesis and tumor progression. The best known example of these genes encodes the cell surface molecule CD44.

CD44 is a transmembrane glycoprotein that mediates the response of cells to their cellular microenvironment. CD44 is expressed in most tissues, where its gene products function in lymphocyte homing, adhesion, migration, and regulation of cell growth [1]. Several functions have been ascribed to some CD44 isoforms. The protein domain coded by exon v3 has been shown to bind growth factors via its heparan sulfate modifications [2], and CD44 v4 is a major E-selection ligand that mediates breast cancer cell transendothelial migration [3]. The variant isoform CD44 v6 has attracted increasing interest since the demonstration 1 decade ago that the transfection of splice variant CD44 v4-v7 conferred metastatic potential on cells of a nonmetastatic rat tumor cell line [4]. The CD44 v6 isoform can form a complex with the extracellular hepatocyte growth factor (HGF) and its tyrosine kinase receptor Met [5]. Formation of this CD44 v6-HGF-Met complex stimulates the activation of Met through autophosphorylation and further activates Met-dependent Ras signalling. The net result is that the CD44 v6-HGF-Met complex activates Ras signalling and promotes cell proliferation. Interestingly, Ras activation also stimulates transcription of CD44 and promotes inclusion of its variable exons [6]. This constitutes a CD44-mediated positive feedback loop in the activation of Ras signalling if a factor such as HGF is present. The tumor suppressor merlin can bind to the cytoplasmic tail of CD44 [7] and disrupts the interaction between ERM and CD44 and therefore inhibits Ras activation. As a result, Ras-dependent CD44 alternative splicing is inhibited, the positive feedback loop is disrupted, and cell growth diminishes [8]. These results raised the possibility that CD44v acts as a prognostic factor of tumor progression and inhibition of CD44 v6 production may be a therapeutic approach of tumor metastasis.

PCBPs are ubiquitously expressed and contain a highly conserved triple repeat of the KH domain. Current data clearly demonstrates that these proteins play important and diverse roles in gene expression at both transcriptional and posttranscriptional levels. PCBP1 (or αCP1 or hnRNP E1), a member of the PCBPs family [9], is widely expressed in many human tissues and involved in regulation of transcription, transportation process, and function of RNA molecules [10]. Moreover, PCBP1 also has been suggested to play a critical role in mRNA splicing. A previous study reported that specific binding of PCBP1 to U1 small nuclear ribonucleoprotein (snRNP) in the pre-spliceosomal complex was associated with silencing of pseudoexon splicing of growth hormone receptor. Another hnRNP family member hnRNP A1 was a component of an exon-specific splice-silencing complex, and transient overexpression of hnRNP A1 prevented CD44v5 splicing. However, the role of PCBP1 in CD44 variants splicing still remains elusive.

In the present study, we provide convincing evidence that human PCBP1 regulates CD44 alternative splicing in human hepatoma cell line HepG2 cells, and loss of PCBP1 in human hepatic tumor may contribute to the formation of a metastatic phenotype.

## Results

### PCBP1 negatively regulates CD44 variants

**Figure 1:**
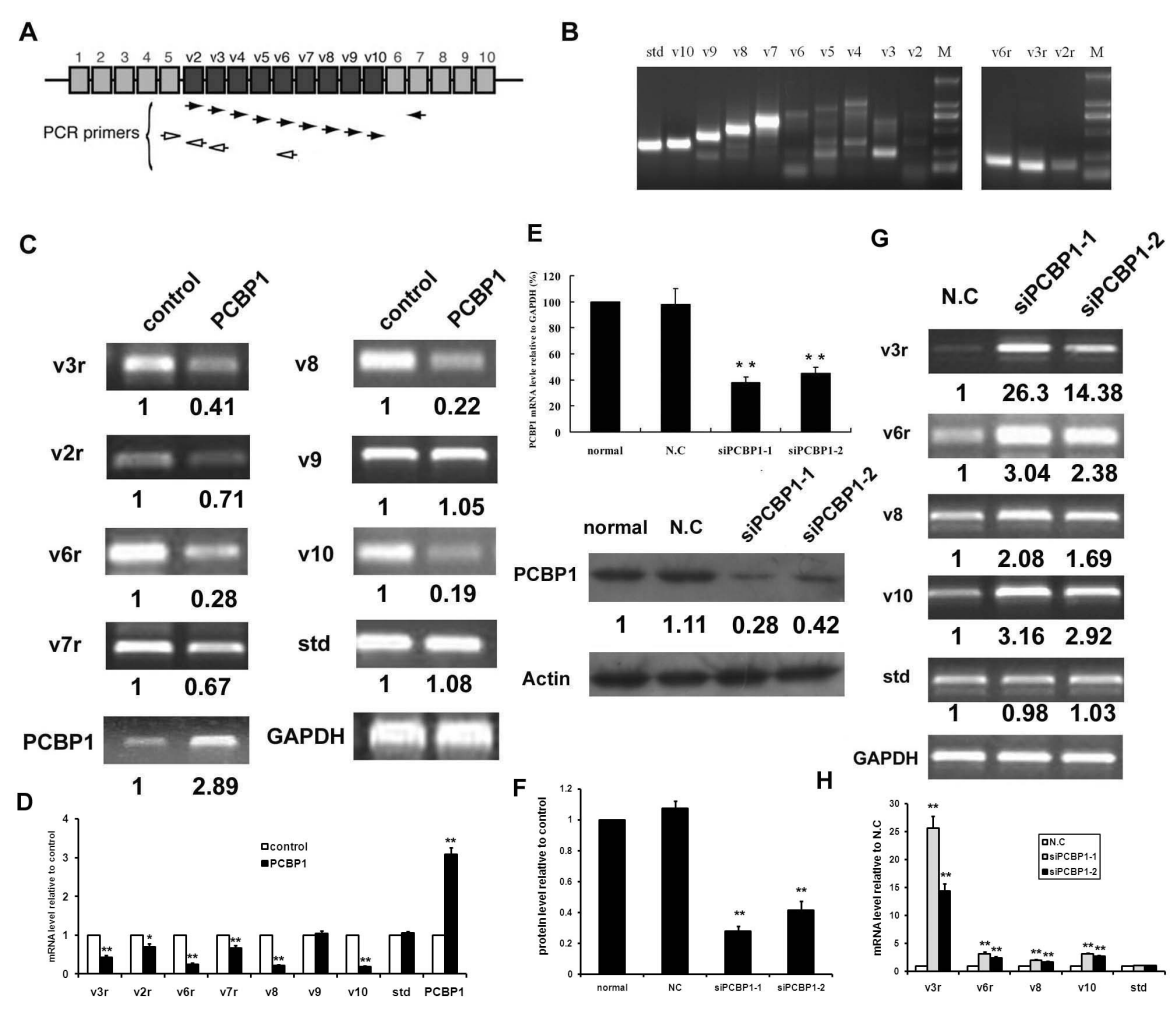
**PCBP1 represses CD44 variants splicing**. (A) Schematic diagram of the primers. (B) Expression profile of CD44 variants in HepG2 cells. (C) pcDNA3.1(His/Myc)-PCBP1 or control vector were transfected into HepG2 cells for 24 hours and total RNA were extracted for semi-quantitative RT-PCR using the primers indicated in (A). The PCR bands were scanned for densitometry analysis with the value obtained from control cells set as 1. The values were normalized with those of GAPDH. Statistical analysis was performed and the results represented mean ± SD of 3 independent experiments. The statistical difference between the samples was demonstrated as *p ≤ 0.05 and ** *p *≤ 0.001 (D). (E) Knockdown of PCBP1. HepG2 cells were transfected with a negative control siRNA (N.C) or PCBP1 specific siRNA oligos (siPCBP1-1 and siPCBP1-2) for 48 hours. The level of PCBP1 was examined by Real-time PCR analysis (upper panel) and Western blotting (bottom panel). Cells with mock transfection were used as control (normal). For Real-time PCR, GAPDH was used as internal control. For Western blotting, the blots were scanned for densitometry analysis with the value obtained from control cells set as 1. The values were normalized with those of Actin. Statistical analysis was performed and the results represented mean ± SD of 3 independent experiments. (F). The levels of CD44 variants were examined by semi-quantitative RT-PCR (G). The PCR bands were scanned for densitometry analysis with the value obtained from N.C cells set as 1 (H).

To investigate whether PCBP1 regulates CD44 variants, HepG2 cells were transfected with pcDNA3.1(His/Myc)-PCBP1 or control vector. A GFP-expression plasmid was cotransfected either with the PCBP1 expressing vector or control vector and all of the transfections displayed a similar efficiency of about 45-48% (data not shown). At 24 hrs after transfection, the levels of CD44 variants were monitored by RT-PCR using the primers described as previously [11] and indicated as Figure 1A. In addition, the CD44 standard form was detected using primers that base-pair to the constitutive exons. The expression profile of CD44 variants in HepG2 cells was shown in Figure 1B. As Figure 1C shows, enforced expression of PCBP1 in HepG2 cells resulted in significant down-regulation of v2, v3, v6, v7, v8, and v10 expression. In contrast, the levels of CD44 v9 and standard form (std) were not affected by overexpression of PCBP1.

To study the effect of endogenous PCBP1 on regulating CD44 alternative splicing, we performed RNA interference assays to investigate whether decreased PCBP1 expression altered CD44 variants expression. To rule out off-target effects, two siRNA oligos were used (siPCBP1-1 and siPCBP1-2). Transfection of HepG2 cells with siPCBP1-1 or siPCBP1-2 resulted in an up to 77% or 44% decrease in the PCBP1 mRNA level (Figure 1D upper panel) and 72% or 58% decrease in the PCBP1 protein level (Figure 1D, bottom panel) within 48 h after transfection. However, transfection with negative control RNA (N.C) had no effect on PCBP1 protein and mRNA level. Furthermore, the levels of β-actin were not affected by negative control siRNAs (Figure 1D). As shown in Figure 1E, when endogenous PCBP1 expression was knocked down by siRNAs, HepG2 cells showed an increased expression of CD44 variants compared to the control cells. To rule out a possible non-specific effect of the negative control siRNA, another negative control siRNA duplex (N.C-2) was used. The similar results were obtained (Figure S1; Additional file 1). So we used N.C as control in the following RNAi experiments. Overall, these results indicated that PCBP1 might play a key role in the regulation of CD44 alternative splicing in HepG2 cells.

**Figure 2:**
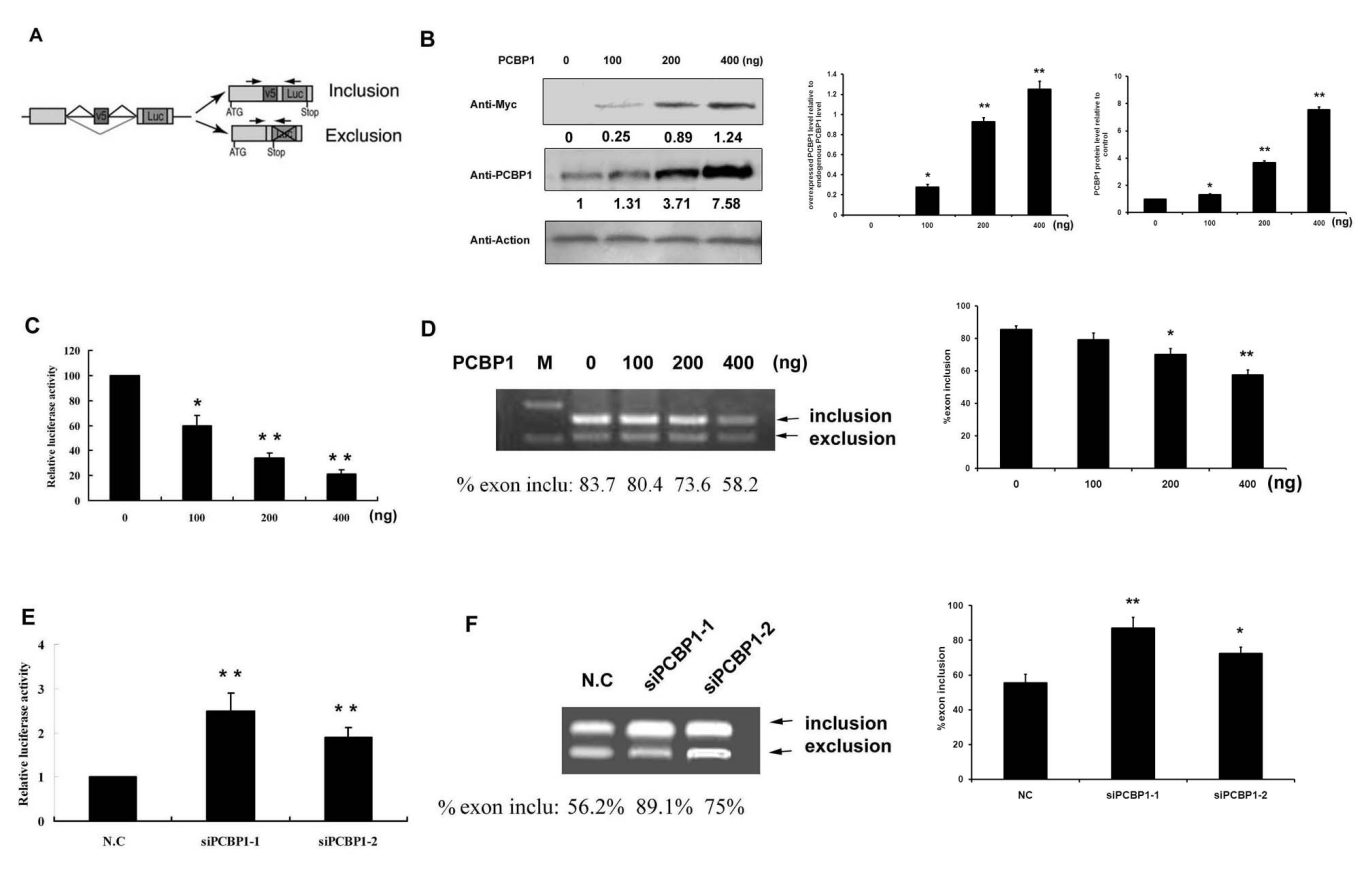
**PCBP1 inhibits CD44 v5 splicing**. (A) Schematic representation of the luciferase splice reporter construct pETCatEBLucv5. (B) The pETCatEBLucv5 vector was cotransfected in HepG2 cells with different doses of pcDNA3.1(His/Myc)-PCBP1 vector and a *Rennila *luciferase plasmid served as an internal transfection control. The expression of overexpressed PCBP1 and endogenous PCBP1 was investigated by Western blotting. The blots were scanned for densitometry analysis with the value obtained from control cells set as 1. The values were normalized with those of Actin. The overexpressed PCBP1 levels were quantified relative to the endogenous PCBP1 expression level. 24 hours later cell lysis were prepared for the luciferase activity assay (C) and total RNA were extracted for RT-PCR (D). The level of luciferase was normalized to 100 following transfection with the control plasmid. The inclusion and exclusion fragments were produced by the primers indicated in (A). Values for exon inclusion were indicated. (E) HepG2 cells were co-transfected with pETCatEBLucv5 vector along with either negative control siRNA (N.C) or PCBP1 specific siRNA oligos for 48 hours and then luciferase activity was measured and the inclusion and exclusion fragments were analyzed by RT-PCR (F). Statistical analysis was performed and the results represented mean ± SD of 3 independent experiments. The statistical difference between the samples was demonstrated as ** *p *≤ 0.001.

We further investigated the role of PCBP1 in the regulation of CD44 v5 expression. Since no specific band was amplified using the present primer pair, we used a CD44 v5 reporter minigene construct pETCatEBLucv5 as described previously [12] (Figure 2A). The pETCatEBLucv5 vector was cotransfected into HepG2 cells with different doses of pcDNA3.1(His/Myc)-PCBP1 indicated as Figure 2B, and a *Rennila *luciferase plasmid was served as an internal transfection control. As Figure 2C shows, PCBP1 overexpression reduced the level of luciferase in a dose-dependent manner. The similar result was obtained by RT-PCR analysis (Figure 2D). Consistently, transient transfection of the PCBP1 siRNA produced an about 2-fold increase of relative luciferase activity (Figure 2E). These results were verified by measuring mRNA levels of v5 inclusion versus exclusion by RT-PCR (Figure 2F).

### PCBP1 inhibits the CD44 v6 expression

**Figure 3:**
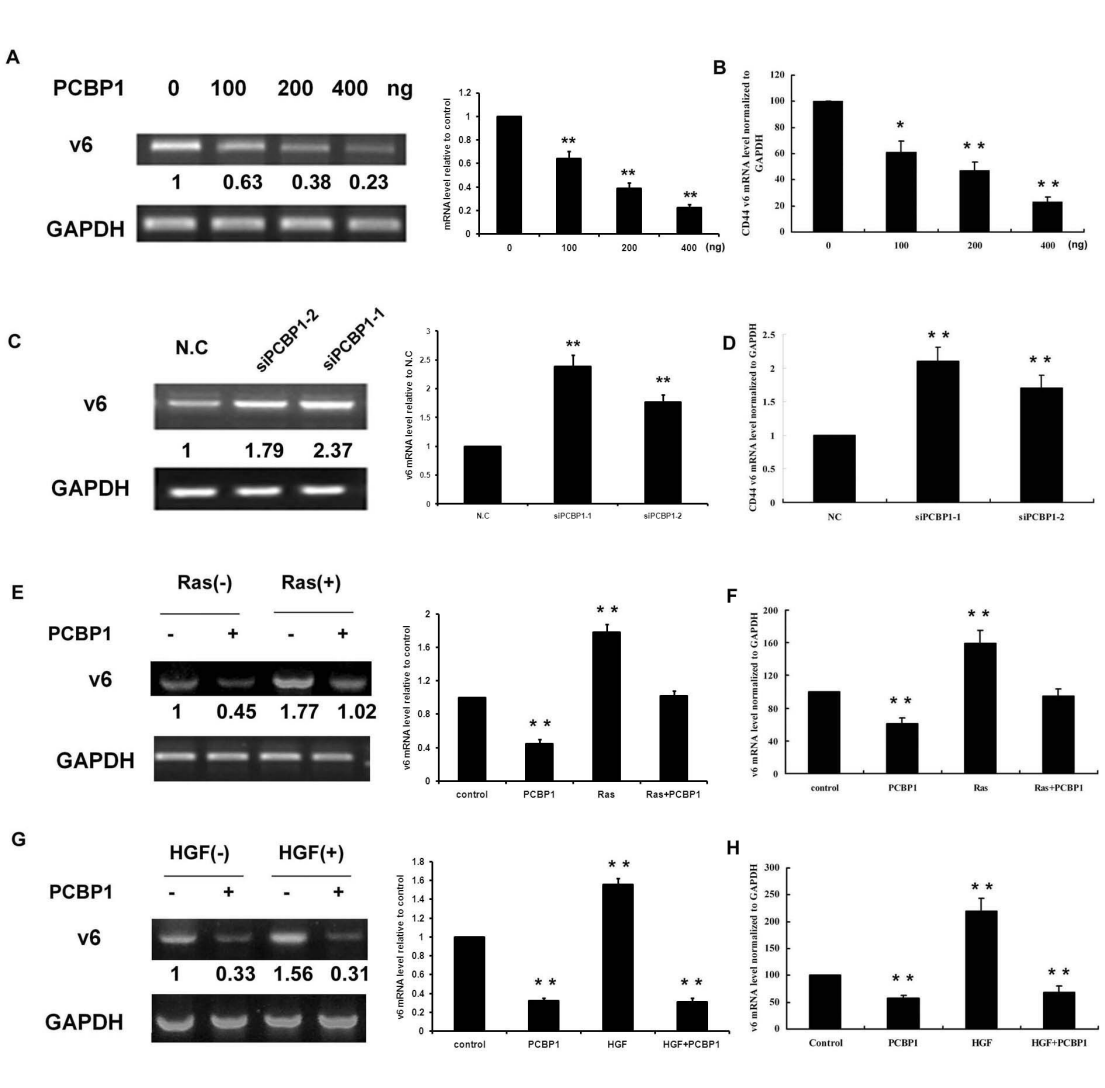
**PCBP1 is a negative regulator of CD44 v6**. (A) Different doses of PCBP1 expression vector as indicated were transfected into HepG2 cells. Cells were harvested 24 hours later and total RNA were extracted for semi-quantitative RT-PCR and Real-time PCR analysis (B) to determine the relative amounts of CD44 v6. (C) HepG2 cells were transfected with a negative control siRNA (N.C) or PCBP1 specific siRNA oligos for 48 hours and then total RNA were extracted. The mRNA level of v6 was examined by semi-quantitative RT-PCR and Real-time PCR analysis (D). (E) PCBP1 inhibits the up-regulation of CD44 v6 induced by Ras activation. HepG2 cells were transfected with a constitutively activated mutant H-Ras V12 construct along with PCBP1 expression vector just as indicated. 24 hours later, total RNA were extracted for semi-quantitative PCR and Real-time PCR analysis (F). (G-H) PCBP1 inhibits the upregulation of CD44 v6 induced by HGF. HepG2 cells were transfected with control vector or PCBP1 expression vector, and then 24 hours later the cells were stimulated with 20 ng/ml HGF for 8 hours. Total RNA was extracted and RT-PCR (G) or Real-time PCR (H) was performed to detect the level of CD44 v6. All the PCR bands were scanned for densitometry analysis with the value obtained from control cells set as 1. The values were normalized with those of GAPDH. Results represented mean ± SD of 3 independent experiments. The statistical difference between the samples was demonstrated as * *p *≤ 0.05 or ** *p *≤ 0.001.

We further investigated the role of PCBP1 in the regulation of CD44 v6 splicing in more detail. HepG2 cells were transfected with different doses of PCBP1 expression vector, and 24 hours later total RNA was extracted for RT-PCR and Real-time PCR analysis. As shown in Figure 3A-B, transiently transfection with increasing amounts of PCBP1 expression vector led to inhibition of CD44 v6 expression in a dose-dependent manner. Moreover, we performed RNA interference assays as described above, and the results suggested that knockdown of endogenous PCBP1 resulted in significant induction of CD44 v6 expression (Figure 3C-D). To confirm the inhibition of CD44 v6 expression by PCBP1, we performed the assays in another hepatocellular carcinoma cell line SMMC-7721 cells and the similar results were obtained (Figure S2; Additional file 1).

We next detected whether PCBP1 inhibits the stimulation of CD44 v6 by Ras/MAPK signalling. A constitutively activated mutant H-Ras V12 construct was co-transfected along with PCBP1 expression vector. As shown in Figure 3E-F, transfection of H-Ras V12 resulted in an approximately 1.6-fold upregulation of CD44 v6 mRNA compared to the control cells. However, co-transfection with PCBP1 expression vector decreased the CD44 v6 expression to basal level.

We further examined whether PCBP1 inhibits the stimulation of CD44 v6 by HGF. HepG2 cells were transfected with control vector or PCBP1 expression vector, and then stimulated with 20 ng/ml HGF for 8 hours. Total RNA were extracted and RT-PCR or Real-time PCR was performed to detect the mRNA level of CD44 v6. As shown in Figure 3G-H, HGF treatment led to about 2.2-fold increase of v6 expression, while overexpression of PCBP1 inhibited the v6 expression significantly. These results confirmed that PCBP1 is an important negative regulator of CD44 v6 expression.

### PCBP1 inhibits HepG2 cell invasion

**Figure 4:**
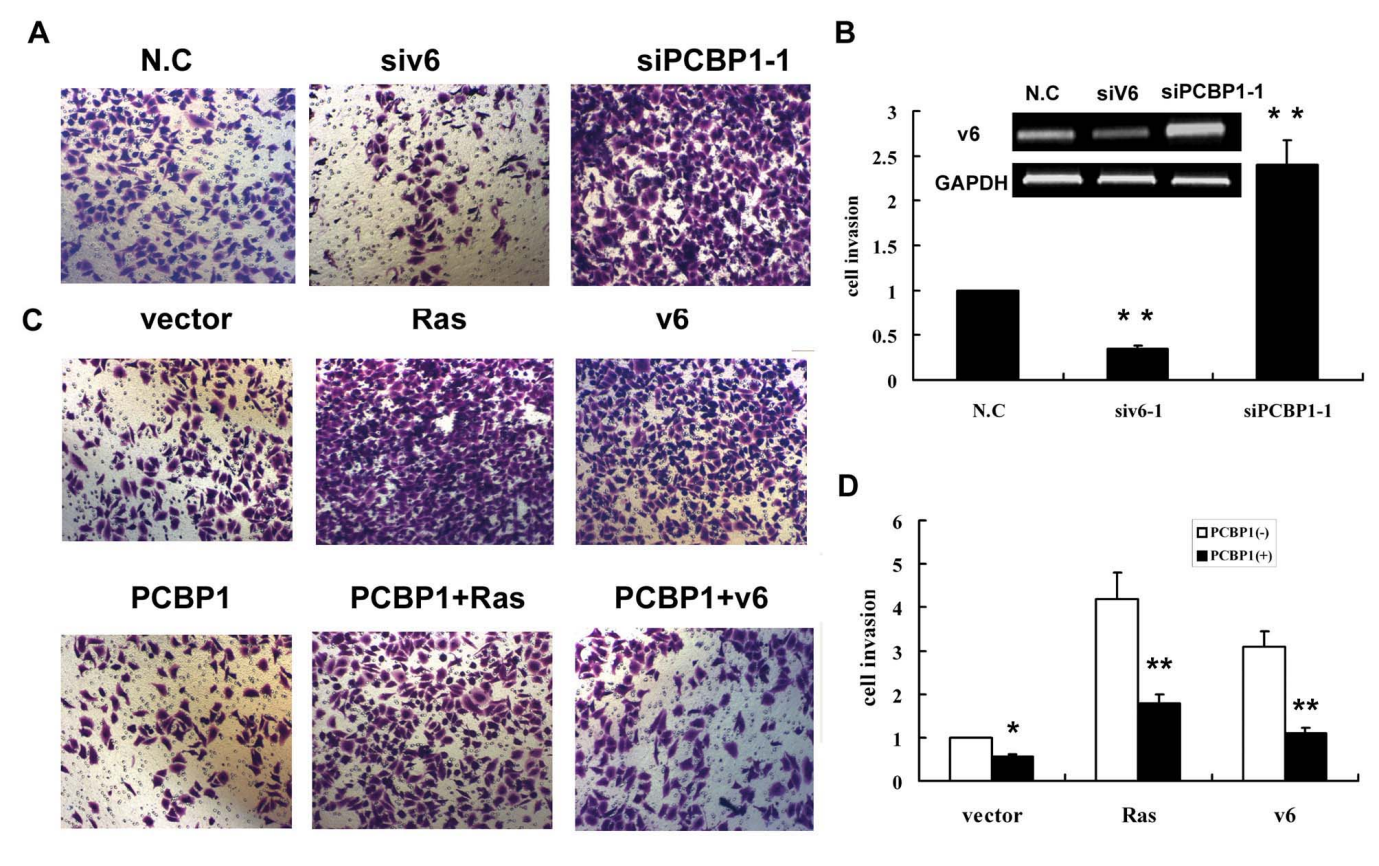
**Negative role of PCBP1 in tumor cell invasion**. (A) Invasion assays of HepG2 cell transfected with negative control siRNA (N.C) or siPCBP1-1 or siv6-1. Quantitation of tumor cell invasion was described in (B). The percentage of cell invasion was normalized to that of cells transfected with control vector. (C) HepG2 cells were transfected with the vectors just as indicated for 24 hours and cell invasion assay was performed. Quantitation of tumor cell invasion was described in (D). The data are mean ± SD of at least three independent studies, each performed in triplicate. The statistical difference between the samples was demonstrated as * *p *≤ 0.05 or ** *p *≤ 0.001.

CD44 v6 have been implicated in tumor invasion and metastasis. Therefore, it is tempting to speculate that the activity of PCBP1 could influence tumor cell invasion by regulating the CD44 v6 expression. We first investigated whether inhibition of CD44 v6 by RNA interference would affect the invasive properties of HepG2 cells. A siRNA direct against v6 exon was transfected into HepG2 cells. As shown in Figure 4A-B, we observed a significant knockdown of v6 in cells treated with v6 siRNA. At 48 h posttransfection, equal numbers of cells treated with either v6 siRNA or control siRNA were added to invasion chambers. After 24 hours of incubation, the cells that invaded through the matrigel membrane and accumulated in the lower chamber were counted. As expected, the number of v6 siRNA-treated cells passing through the membrane was only approximately 35% of the control siRNA-treated cells. We observed similar results when the above-described experiments were repeated using a second v6 siRNA (data not shown). These results demonstrated that CD44 v6 expression plays a key role in HepG2 cell invasion.

To detect the effect of PCBP1 in cell invasion, PCBP1 expression vector or control vector was transfected into HepG2 cells and cell invasion was measured. The number of PCBP1 transfected cells passing through the membrane was only approximately 55% of the control cells (Figure 4C-D). However, knockdown of PCBP1 in HepG2 cells led to about 2.4-fold increased in the number of cells passing through the membrane. These results suggested that PCBP1 was involved in regulation of HepG2 cell invasion. The similar results were obtained in SMMC-7721 cells (Figure S3; Additional file 1).

We further investigated the relationship of PCBP1 and v6 on tumor cell invasion. HepG2 cells were cotransfected with PCBP1 expression vector and a Ras expression vector or a CD44 v6 expression construct. As shown in Figure 4C-D, overexpression of Ras or v6 expression vector led a significant increase of v6 expression (data not shown) and cell invasion (Figure 4C-D). Cells transfected with Ras or v6 expression vector showed about 4.2-fold or 3.1-fold increase of cell invasion respectively. Furthermore, re-expression of CD44 v6 by Ras activation or v6 expression vector in cells transfected with PCBP1 attenuated the inhibition of cell invasion by PCBP1. Ras activation in PCBP1-transfected cells led to about 1.8-fold increase of cell invasion while v6 expression vector reversed the inhibition of cell invasion by PCBP1 to a level similar to the control. These results suggested that PCBP1 could play an important role in tumor cell invasion by altering the level of CD44 v6 in HepG2 cells.

### Up-regulation of CD44 v6 expression is accompanied by down-regulation of PCBP1 in HCC

**Figure 5:**
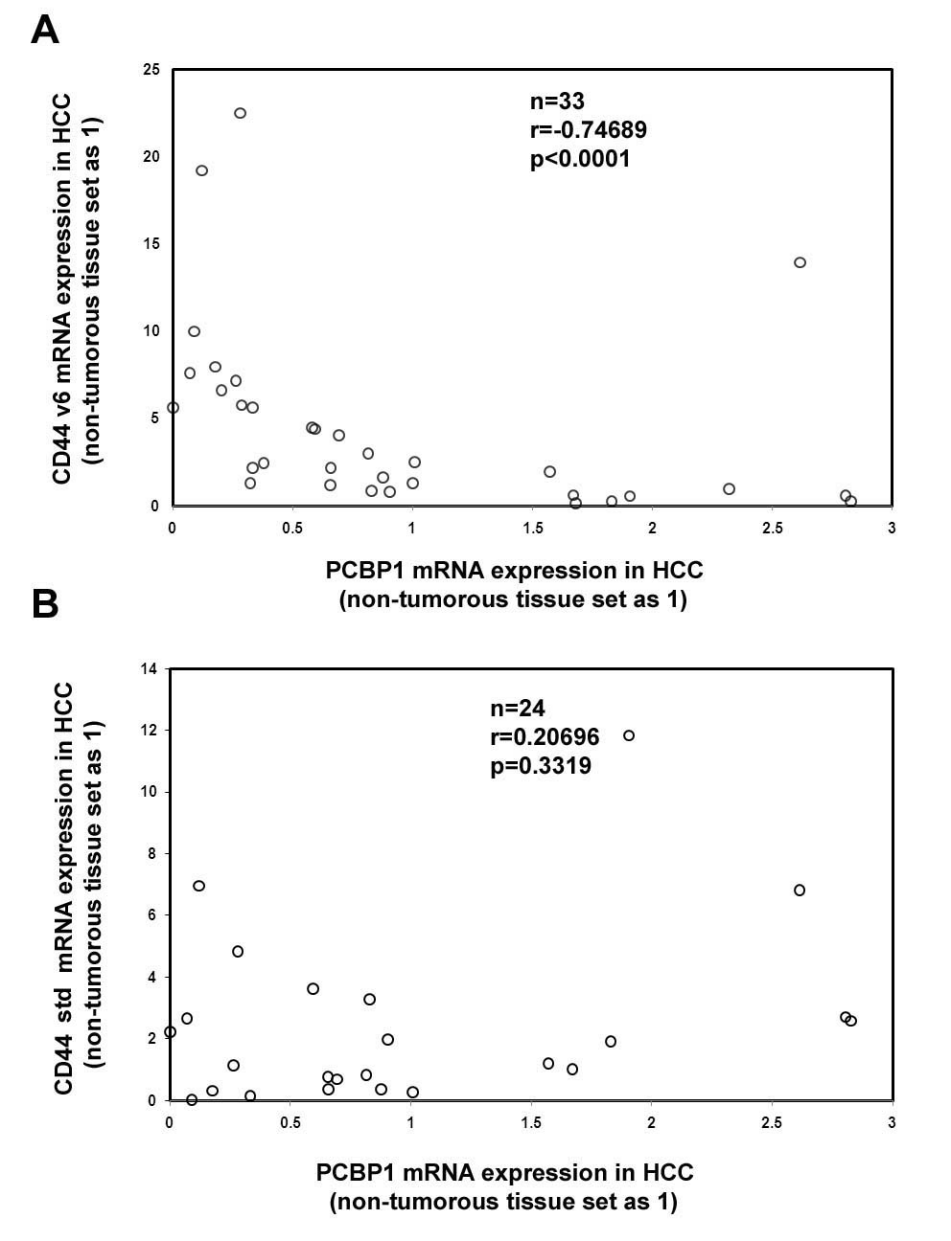
**Negative Correlation of PCBP1 and CD44 v6 mRNA expression in HCC tissue samples analyzed**. Total RNA was extracted from HCC specimens and their adjacent non-cancerous liver tissues for real-time PCR analysis. The expression levels of PCBP1, v6 and std in non-cancerous tissues were set to 1 and normalized to GAPDH mRNA. The experiments in each sample were performed in triplicate with at least 3 independent times. The data was shown as the average of these experiments. Then Spearman test was performed.

Since PCBP1 was a negative regulator of CD44 v6 expression and tumor invasion, we further examined whether a correlation of the expression status of PCBP1 and CD44 v6 exists in primary HCCs. Real-time PCR analysis was performed to detect the mRNA levels of PCBP1 and CD44 v6 in HCC tissue and the adjacent non-cancerous liver tissues of 33 HCC patients. The result suggested that in HCC tissues, PCBP1 showed significant negative correlation with the expression of v6 (n = 33, r = -0.74689 p < 0.0001) (Figure 5A), that is, in HCC patients which PCBP1 was down-regulated compared to the non-cancerous liver tissue, the CD44 v6 expression level showed a significant up-regulation. However, no significant correlation was detected between PCBP1 expression and CD44 std expression in HCC (n = 24, r = 0.20696, p = 0.3319) (Figure 5B). These data suggested that the reduction of PCBP1 expression correlated with the up-regulation of CD44 v6 expression in HCC.

## Discussion

In the present study, we first characterized PCBP1 as a novel negative regulator of CD44 variants splicing. Ectopic expression of PCBP1 inhibits tumor invasion and knockdown of endogenous PCBP1 expression stimulates invasion, suggesting a negative role of PCBP1 in tumor metastasis.

Alternative pre-mRNA splicing is controlled by coordinate regulation between positive and negative factors. Various studies suggest that alternative splicing can be regulated by both *cis*-acting RNA elements, such as exonic splicing enhancers (ESEs) and exonic splicing silencers (ESSs), and *trans*-acting factors that can bind to RNA elements directly. Two groups of proteins have been found that can affect splice site choice: serine/arginine-rich (SR) proteins and heterogeneous nuclear ribonucleoproteins (hnRNPs) [13]. ESEs are often bound by SR proteins whereas exonic splicing silencers ESSs are typically bound by hnRNP proteins [14]. A previous study report that specific binding of PCBP1 to U1 small nuclear ribonucleoprotein (snRNP) in the pre-spliceosomal complex was associated with silencing of pseudoexon splicing of growth hormone receptor. Moreover, mitogenic stimulation promotes Pak1- and PCBP1-dependent alternative splicing and exon inclusion from a CD44 minigene. The alternative splicing functions of PCBP1 are in turn mediated by its intrinsic interaction with Caper alpha, a U2 snRNP auxiliary factor-related protein previously implicated in RNA splicing. Furthermore, another hnRNP family member hnRNP A1 was a component of an exon-specific splice-silencing complex, and transient overexpression of hnRNP A1 prevented v6 splicing. These results indicate that PCBP1 might be a trans-acting factor in silencing of mRNA splicing just like hnRNP A1 does. In our present study, we confirmed that PCBP1 inhibits CD44 variant splicing including v5 and v6 and knockdown of endogenous PCBP1 stimulates the CD44 variants splicing, suggesting that PCBP1 is a novel negative regulator of CD44 variants splicing. However, in a previous study [15], a positive role of PCBP1 in CD44 v5 splicing was detected. This difference might be due to the different cell lines used and a phosphorylation of PCBP1 by Pak-1 in the presence of EGF in their cell models.

Variant CD44 isoforms are involved in a variety of physiological and pathological processes, among which tumor progression has received most attention. During the past decade, a vast number of primary tumors and metastases from close to 10,000 patients have been screened for the expression of CD44 v6. This expression pattern has made CD44v6 an attractive target for antibody-guided therapy of various types of cancers [16]. Our data in the present study suggest a novel function of PCBP1 in the regulation of CD44 v6 splicing. Previous studies suggest that the activation of Ras/MAPK signaling induced by HGF initiates a positive feedback loop by stimulating isoform-specific CD44 expression through alternative splicing. This positive feedback loop plays a key role in the transition from normal to transformed phenotypes. PCBP1 could inhibit the v6 expression induced by Ras activation and HGF and then break the positive feedback loop, suggesting that PCBP1 might function as a candidate tumor suppressor and play a potential role in the gene therapy of malignant tumors with v6 overexpression. Furthermore, we demonstrate the association between up-regulation of v6 and down-regulation of PCBP1 in primary HCC patients, which raises the possibility that v6 overexpression in liver cancer may be partly due to the down-regulation of PCBP1. Consistent with these observations, overexpression of PCBP1 in HepG2 cells dramatically inhibits the v6 expression and tumor invasion while re-expression of v6 reverses the effect of PCBP1 in cell invasion. Thus, it is possible that PCBP1 regulates the cell invasion partly through regulating the CD44 v6 expression level.

Interestingly, our data suggest that PCBP1 regulates the CD44 alternative splicing in an exon-specific manner. PCBP1 inhibits the splicing of v3, v5, v6, v7 and v8, while has no effect on v9, and standard form of CD44. PCBP1 has three KH domains and the first and the second KH domains can independently bind poly(rC) with a high affinity and specificity [17]. So it seems that PCBP1 regulates the CD44 alternative splicing by binding to mRNA directly. However, sequence comparison of the CD44 variant exon v3 through v8 revealed no obvious common exon silencing elements or the consensus sequence binding to PCBP1, indicating that PCBP1, possibly in association with other partner proteins, through an exon-specific combination of distinct mall sequence elements in a cooperative manner. Recently, hnRNP proteins have been shown to hinder communication between factors bound to different splice sites. Moreover, cooperative interactions between bound hnRNP proteins may encourage splicing between specific pairs of splice sites while simultaneously hampering other combinations. Thus, hnRNP proteins utilize a variety of strategies to control splice site selection in a manner that is important for alternative pre-mRNA splicing [18]. Further identification of PCBP1 binding partners may facilitate understanding of the mechanism of PCBP1 in selectively regulating mRNA splicing.

Although the functions of PCBPs in alternative mRNA splicing, translation silencing and transcriptional regulation have been widely reported, only a few studies suggest that PCBP1 might play a role in tumor invasion. In a previous study, analysis of PCBP1 expression in different grade squamous intraepithelial lesions, and invasive cervical carcinoma showed that PCBP1 expression decreased in high grade squamous intraepithelial lesions and cancer, respectively and the loss of PCBP1 expression may allow HPV infected cervical tissues to undergo virus mediated transformation from cervical dysplasia to cancer [19]. Various studies have suggested that several other members of the hnRNP family associate with tumor. The pre-mRNA processing protein, hnRNP A2/B1, has been proposed as a promising biomarker for lung cancer [20]. The overexpression of hnRNP C1/C2 was demonstrated in HCC samples, suggesting their potential as protein tumor markers [21]. In addition, hnRNP F is down-regulated in HCC and up-regulated in gastric carcinoma, indicating the important while complex potential role of this subset of hnRNPs on the gene expression in different tissues [22]. Furthermore, the PCBP1 gene maps to human chromosome 2 (2p13-12) which is one of the most frequently altered region in human malignancies. Pesti et al also delineated this region as a tumor suppressor gene region [23]. In the present study, we provide convincing evidence that PCBP1 is a negative regulator of cell invasion in HepG2 cells.

## Conclusions

Taken together, our present study suggests a negative role of PCBP1 in CD44 variants splicing and cell invasion in HepG2 cells, which raises the possibility that down-regulation of PCBP1 might be a biomarker in tumor metastasis.

## Methods

### Tissue specimens

All human hepatocellular carcinoma (HCC) specimens were obtained from those patients who underwent surgical resection of their diseases and were informed consent before operation on their liver. The primary tumor specimens were immediately frozen at -80°C until RNA extraction. Both tumor and adjacent nontumor tissues were sampled respectively, with approximate 1 cm^3 ^size of each specimen, and were proved by pathological examination. This study was approved by the ethics committee at the Beijing Institute of Radiation Medicine,

### Cells and transfection

Human hepatoma cell lines HepG2 and SMMC-7721 cells were purchased from the Cell Institute of Chinese Academy of Science (Shanghai, China) and maintained in Dulbecco's modified Eagle's medium (Gibco) supplemented with 10% fetal bovine serum (Hyclone) at 37°C in 5% humidified CO_2_. DNA transfection was carried out using Lipofectamine2000 (Invitrogen, Carlsbad, CA, USA) as a mediator according to the manufacturer's instruction.

### Plasmid constructions

The human full-length PCBP1 were amplified by PCR from the human liver cDNA and cloned into the pcDNA3.1 (His/Myc) vector (Invitrogen, Carlsbad, CA, USA). pETCatEBlucv5 was kindly provided by Prof. Weg-Remers S (Forschungszentrum Karlsruhe der Helmholtz-Gemeinschaft, Institut für Toxikologie und Genetik, Karlsruhe, Germany) The constitutive activation H-Ras V12 construct was kindly provided by Dr. Herb Sun (Mount Sinai School of Medicine, New York, USA). The sequences of the primers are provided in Table S1; Additional file 2.

### RNA interference (RNAi)

The small interfering RNA (siRNA) oligos of PCBP1 were synthesized in GenePharma Biotechnology, the sequences are as follows: si-PCBP1-1, sense: CAC CAU UCC AAA UAA CUU ATT; antiense: UAA GUU AUU UGG AAU GGU GAG; si-PCBP1-2, sense: GAA CCA GGU GGC AAG ACA ATT; antisense: UUG UCU UGC CAC CUG GUU CAG. The sequence of CD44 v6 siRNA oligos are: si-v6-1, sense: UGA GGG AUA UCG CCA AAC ATT; antisense: UGU UUG GCG AUA UCC CUC ATT; si-v6-2, sense: GCA ACU CCU AGU AGU ACA ATT; antisense: UUG UAC UAC UAG GAG UUG CTT. The negative control siRNA (N.C) was obtained from Qiagen (Qiagen, Germantown, MD, USA). Another negative control siRNA (N.C-2) was obtained from GenePharma (GenePharma, Shanghai, China) with different sequence from N.C: sense: UUC UCC GAA CGU GUC ACG UTT; antisense: ACG UGA CAC GUU CGG AGA ATT. siRNA oligos were transfected into HepG2 cells using Lipofectamine 2000 (Invitrogen) at a concentration of 20 nM.

### Transfection and luciferase assay

Cells were plated in 24-well tissue culture plate at a density of 5 × 10^4^cell per well and transfected with 0.5 μg of each construct reporter plasmid. As an internal control, the pRL-TK vector was cotransfected which led to the constitutive expression of *Rennila *luciferase (Promega Corp., Madison, WI, USA). Twenty four hours after transfection, luciferase activity was measured with the Dual Luciferase Assay system (Promega) according to the manufacturer's instructions. Briefly, the cells were lysed directly in the 24-well plate using 100 μl of 1 × passive lysis buffer provided with the Dual-Luciferase kit. One hundred microliters of LARII reagent (firefly luciferase substrate) was added to 50 μl of lysis supernatant, and firefly luciferase activity was measured with a chemiluminescence analyzer (FB12 luminometer; Berthold Detection Systems, Montreal Biotech Inc., CA, USA) for 10 s. Then, 100 μl of Stop-n-Glo reagent (*Renilla *luciferase substrate) was added to the reaction, and *Renilla *luciferase activity was measured for an additional 10 s. Firefly luciferase activities were normalized by *Renilla *activities to correct for differences in transfection efficiencies. All transfection and reporter assays were performed independently at least three times. Firefly luciferase activities normalized by *Renilla *activities are presented as fold induction relative to the normalized firefly luciferase activity in cells transfected with empty vector only or negative control siRNA oligos, which was taken as 100% or 1.0.

### Reverse transcription-PCR (RT-PCR) and quantitative real-time RT-PCR

Total RNA was reverse-transcribed and amplified using reverse transcription (RT; Promega) and PCR (Promega) kits, respectively. The PCR products were electrophoresed on 1% agarose gels and photographed under ultraviolet light. Quantity One software (BioRad, USA) was used for densitometry analysis. Quantitative real-time RT-PCR was done by ABI Prism 7700 Sequence Detector (Applied Biosystems, Foster City, CA). The abundance of mRNA of each gene was normalized to the amount of total RNA. The sequences of the primers are provided in Table S1; Additional file 2.

### Western blotting

For Western blotting, cells were lysed with M-PER^® ^Mammalian Protein Extraction Reagent (Pierce, Rockford, IL). Then, Western blot analysis was performed according to standard procedures. Antibodies were used at the following concentrations: anti-Myc, 1:500 (Santa Cruz Biotechnology, Santa Cruz, CA; anti-PCBP1, 1:500 (Santa Cruz) and anti-β-actin (sc-47778, Santa Cruz), 1:1000. Chemiluminescent detection was conducted using supersignal substrate, according to the manufacturer's specifications.

### Matrigel invasion assay

Biocoat Matrigel invasion chambers (Becton Dickinson) were used to assess the invasiveness of HepG2 cells. 5 × 10^4 ^cells were resuspended in 250 μl of serum-free DMEM and added to the cell culture inserts of the invasion chambers. Fetal bovine serum (10%) was used as a chemoattractant and placed in the lower wells. After 22 to 24 h, cells on the upper surface of the membrane were removed using cotton swabs, and the filters were fixed by immersion in 4% formaldehyde for 10 min. After two washes with water, the invaded cells were then stained with 0.2% crystal violet. Excess dye was rinsed off two times with water. The number of cells that had migrated to the lower surface of the filter membrane was counted in five randomly chosen fields under a light microscope

### Statistical Analysis

All experiments were performed at least three times. Data were reported as means ± SD and a value of *p *≤ 0.05 was considered to be statistically significant. Students-Newman-Keuls tests were used to analyze comparisons between groups. The Spearman correlation test was used to analyze the correlation between parameters.

## Abbreviations

HGF/SF: hepatocyte growth factor/Scatter factor; HCC: human hepatocellular carcinoma; PCBPs: poly (rC)-binding proteins; PCR: polymerase chain reaction; hnRNP: heterogeneous nuclear ribonucleoproteins.

## Competing interests

The authors declare that they have no competing interests.

## Authors' contributions

TZ performed luciferase assay and RT-PCR analysis. X-HH performed siRNA knock-down. LD performed the RNA extraction from primary HCC tissues. DH constructed the PCBP1 expression vector. Y-QZ performed cell culture. W-XX performed bacterial culture and purification of the plasmid. XW participated in PCR analysis and data collection. LT performed the Real-time PCR analysis. CG, MY and WL participated in discussion and manuscript preparation. C-YL designed experiments, drafted manuscript and performed cell invasion. X-MY conceived the study and revised the manuscript. All authors read and approved the final manuscript.

## Supplementary Material

Additional file 1 **Additional Figures**. Additional Figures containing Figure S1-S3. Click here for file

Additional file 2 **Table S1**. All the sequences of primers used in Real-time PCR and semi-quantitative RT-PCR analysis. Click here for file
