# Supplementary material for: PCBP-1 regulates alternative splicing of the CD44 gene and inhibits invasion in human hepatoma cell line HepG2 cells
Source: Mol Cancer. 2010 Apr 2;9:72. doi: 10.1186/1476-4598-9-72 (PMC2864215; doi:10.1186/1476-4598-9-72)
Supplement: Additional file 1 — Additional Figures. Additional Figures containing Figure S1-S3. [file 1476-4598-9-72-S1.DOC]

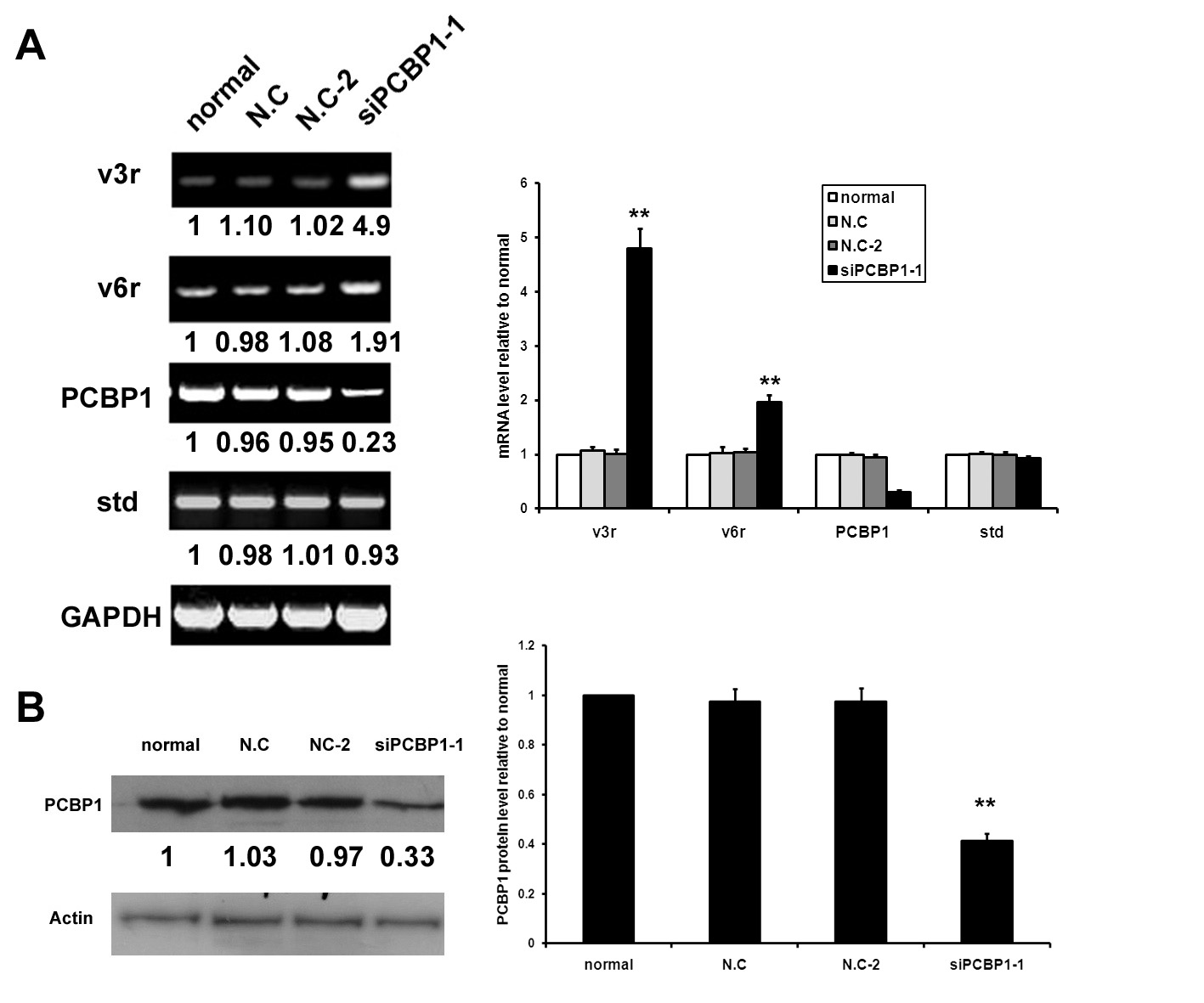


**Figure S1 N.C-2 has no non-specific effects on PCBP1 and CD44 variants expression.** HepG2 cells were transfected with negative control siRNA (N.C or N.C-2) or PCBP1 specific siRNA oligos (siPCBP1-1) for 48 hours and then total RNA were extracted. The levels of CD44 variants and PCBP1 were examined by semi-quantitative RT-PCR (A) and the PCBP1 protein level was investigated by Western blotting (B). Cells with mock transfection were used as control (normal). For semi-quantitative RT-PCR, the bands were scanned for densitometry analysis with the value obtained from normal cells set as 1. The values were normalized with those of GAPDH. For Western blotting, the bands value obtained from normal cells set as 1 and normalized with those of Actin. Statistical analysis was performed and the results represented mean ± SD of 3 independent experiments. The statistical difference between the samples was demonstrated as ** *p* ≤0.001.


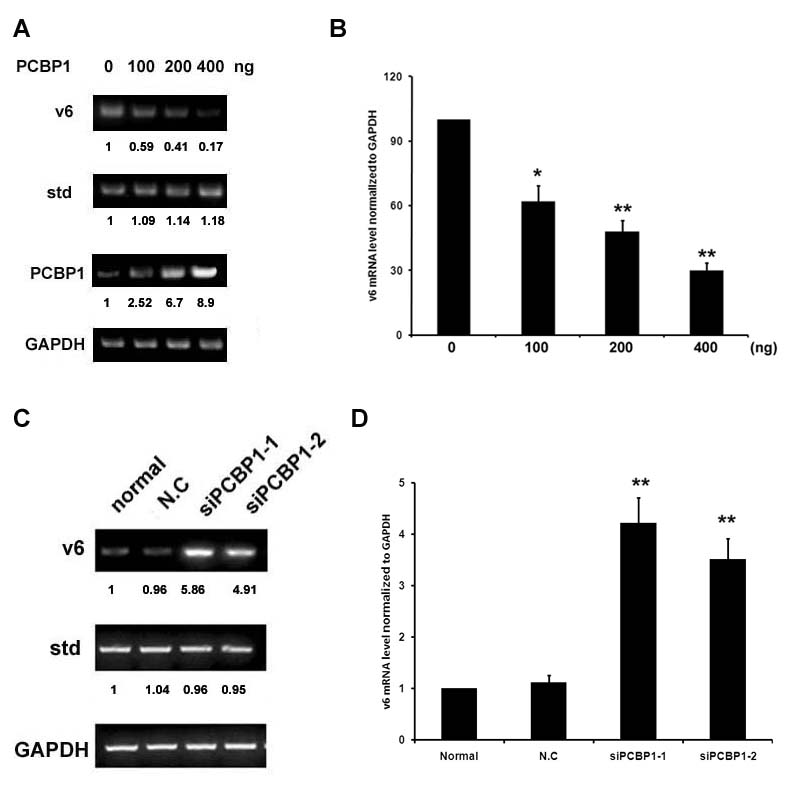


**Figure S2** **PCBP1 inhibits CD44 v6 expression in SMMC-7721 cells.** (A) Different doses of PCBP1 expression vector as indicated were transfected into SMMC-7721 cells. Cells were harvested 24 hours later and total RNA were extracted for semi-quantitative RT-PCR(A) and Real-time PCR analysis (B) to determine the relative amounts of CD44 v6. (C) SMMC-7721 cells were transfected with a negative control siRNA (N.C) or PCBP1 specific siRNA oligos for 48 hours and then total RNA were extracted. The mRNA level of v6 was examined by semi-quantitative RT-PCR and Real-time PCR analysis (D) to determine the relative amounts of CD44 v6. The PCR bands were scanned for densitometry analysis with the value obtained from control cells set as 1. The values were normalized with those of GAPDH. The bands shown are representatives of four independent experiments. For Real-time PCR analysis, results represented mean ± SD of 3 independent experiments. The statistical difference between the samples was demonstrated as * *p* ≤ 0.05 or ** *p* ≤0.001.


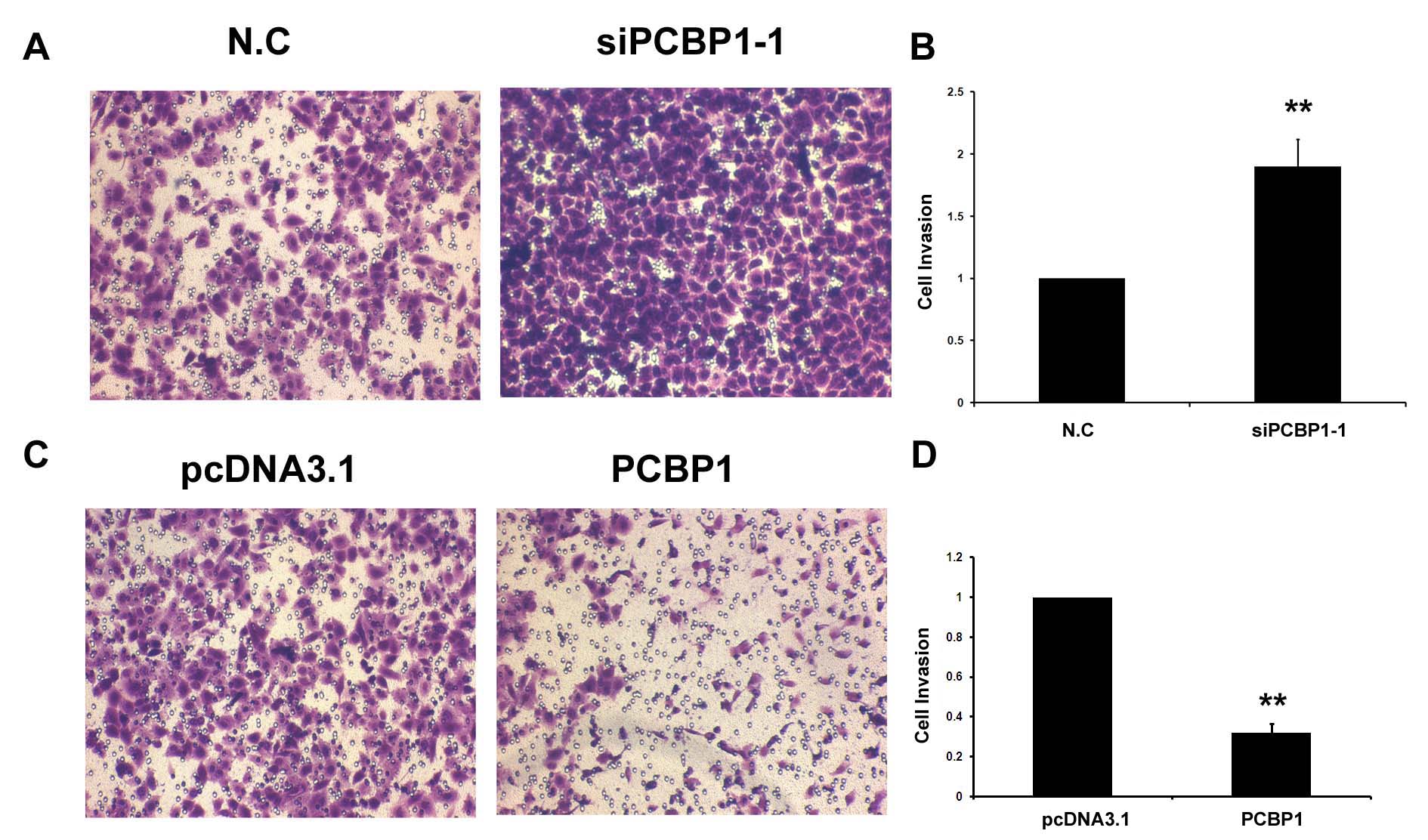


**Figure S3 Negative role of PCBP1 in cell invasion of SMMC-7721 cells.** (A) Invasion assays of SMMC-7721 cell transfected with negative control siRNA (N.C) or si PCBP1-1. Quantitation of tumor cell invasion described in (B). The percentage of cell invasion was normalized to that of cells transfected with control vector. (C) SMMC-7721 cells were transfected with pcDNA3.1 or PCBP1 expression vector for 24 hours and cell invasion assay was performed. Quantitation of tumor cell invasion described in (D). The data are mean ± SD of at least three independent studies, each performed in triplicate. The statistical difference between the samples was demonstrated as ** *p* ≤0.001.
