# Supplementary material for: PCBP-1 regulates alternative splicing of the CD44 gene and inhibits invasion in human hepatoma cell line HepG2 cells
Source: Mol Cancer. 2010 Apr 2;9:72. doi: 10.1186/1476-4598-9-72 (PMC2864215; doi:10.1186/1476-4598-9-72)
Supplement: Additional file 2 — Table S1. All the sequences of primers used in Real-time PCR and semi-quantitative RT-PCR analysis. [file 1476-4598-9-72-S2.DOC]

| primer | Sequence (5’-3’) | target | Use |
| --- | --- | --- | --- |
| PCBP1 F | CG GAA TTC ATG GAT GCCGGTGTGACTGAA | PCBP1 | Construct into pcDNA3.1(His/Myc) |
| PCBP1 R | CGCCTCGAGGCGCTGCACCCCATGCCCTTC | PCBP1 | Construct into pcDNA3.1(His/Myc) |
| V5 F | GAGGGATCCGCTTCCTGCCCC | CD44 V5 | v5 inclusion/exclusion |
| V5R | CCA GCG GAT AGA ATG GCG CCG | CD44 V5 | v5 inclusion/exclusion |
| STD F | AAGACATCTACCCCAGCAAC | CD44 Standard | CD44s |
| V R | TTTGCTCCACCTTCTTGACTCC | CD44 Standard | CD44s, variants reverse primer |
| V2 F | GATGAGCACTAGTGCTACAG | CD44v2 | CD44v2 |
| V3 F | ACGTCTTCAAATACCATCTC | CD44v3 | CD44v3 |
| V4 F | TCAACCACACCACGGGCTTT | CD44v4 | CD44v4 |
| V5F | GTAGACAGAAATGGCACCAC | CD44v5 | CD44v5 |
| V6F | GAGGCAACTCCTAGTAGTAC | CD44v6 | CD44v6 |
| V7F | CAGCCTCAGCTCATACCAGC | CD44v7 | CD44v7 |
| V8F | TCCAGTCATAGTACAACGCT | CD44v8 | CD44v8 |
| V9F | CAGAGCTTCTCTACATCACA | CD44v9 | CD44v9 |
| FV10 | GGTGGAAGAAGAGACCCAAA | CD44v10 | CD44v10 |
| VRF | CATCCCAGACGAAGACAGTC |  | V2r,v3r, v6r, v7r forward primer |
| V2r R | TGTGAAGATGATTCTTTGACTC | CD44v2 | CD44v2r |
| V3r R | CATCATCAATGCCTGATCCAGA | CD44v3 | CD44v3r |
| V6r R | CAGCTGTCCCTGTTGTCGAA | CD44v6 | CD44v6r and Real-time PCR |
| V7r R | TCCTGCTTGATGACCTCGTC | CD44v7 | CD44v7r |
| GAPDH qF | AACGTGTCAGTGGTGGACCT | GAPDH coding region | Real-time PCR |
| GAPDH qR | TGCTGTAGCCAAATTCGTTG | GAPDH coding region | Real-time PCR |
|  |  |  |  |

**Table S1. Sequences of primers used in the present study**
